# Integration of a large language model with augmentative and alternative communication tool for oncological aphasia rehabilitation

**DOI:** 10.1016/j.apjon.2023.100344

**Published:** 2023-11-18

**Authors:** Yingchun Zeng, Qi Tang, Shuchang Chen, Xiaoqin Chen

**Affiliations:** a Affiliated Hospital, Hangzhou City University, Hangzhou, China; b School of Medicine, Hangzhou City University, Hangzhou, China

Aphasia is a neurogenic language disorder in which a person is unable to understand spoken language, produce it, or both.^1^ Oncological aphasia is caused by brain tumours and their surgical treatment that result in damage to the language centres of the cerebral cortex in the dominant hemisphere, which affects language comprehension or speech expression.^2^ Aphasia is common in those with brain tumors; about 30% to 50% of patients with primary brain tumors experience aphasia.^3^ Oncological aphasia caused by brain tumors is one of the most prominent neurological impairments, and the prevalence ranges up to 48% of patients.^4^

Oncological aphasia is an extremely distressing problem that seriously harms patients’ daily lives and physical and mental health. People with oncological aphasia experience a lower health-related quality of life, with reduced activity and independence resulting in social isolation, a lower quality of social relationships, and limited accessibility.^5^, ^6^, ^7^ Because oncologic aphasia can negatively affect well-being and quality of life, seeking effective language function recovery technology has become an urgent need for patients and a key focus of current rehabilitation. This editorial aimed to propose the integration of a large language model (LLM) (Generative Pre-Trained Transformer 4 [GPT-4]) with the augmentative and alternative communication (AAC) tool for oncological aphasia rehabilitation.

Health care providers usually manage oncological aphasia by actively promoting the return of language function and avoiding iatrogenic damage. A key to promoting language recovery is to provide intensive language training, which lasts several hours per week.^8^^,^^9^ Previous studies have found that intense treatment over a short period is more efficacious than low-intensity therapy over a longer period.^10^ Traditional rehabilitation is mainly performed by specialised rehabilitation practitioners, and not all patients can afford the costs associated with these private health care services.^11^ In addition, traditional rehabilitation regimes tend to be monotonous and repetitive, and patient compliance is relatively low. Therefore, novel rehabilitation methods that act as an alternative or adjunct to traditional approaches are urgently needed to maximise the language recovery process.

Recently, many innovations have been introduced as potential language rehabilitation approaches, especially virtual reality (VR) technology. VR technology can create interactive computer mediums incorporating visual, aural, and tactile simulations.^12^^,^^13^ These applications provide computer-simulated immersive scenarios that allow people with communication disorders to enter language-simulated scenarios of varying levels of complexity, offering the opportunity to interact socially and practice daily communication.^14^ VR technology enhances the ecological validity of language therapy by utilising virtual environments that mimic real-life environments. Such technology has been proven to offer obvious advantages. For example, virtual therapies in VR require little or no input from a practitioner in real time, which eases clinicians' workload and reduces patients’ cost of treatment.^15^ Another clear advantage of the technology is that it allows patients to practice anytime and anywhere, thus facilitating intensive treatment.^16^

In contrast to repetitive training tasks in traditional rehabilitation regimes, which lead to difficulty maintaining patients’ interests and engagement, a range of training tasks in VR systems can be designed in advance, again promoting compliance. Furthermore, there are non-language deficits associated with aphasia, such as prosody and emotional tone, that could also be addressed with VR.^13^ This has yet to be incorporated into this approach. A current limitation is that the wearing and use of VR devices may not be suitable for patients who have recently undergone brain tumor surgery.

Considering these limitations, SocializeChat, as an emerging AAC tool, may become a key option for language rehabilitation therapy in patients with oncological aphasia in the future.^17^ This system combines eye gaze tracking with a LLM to improve social language training. Firstly, SocializeChat's interface utilises eye gaze tracking input, with previous research focusing on maximising entry rates, allowing users of AAC tools to achieve text input rates similar to those of able-bodied people.

In addition to SocializeChat's refined input speed, it also uses an LLM to generate content that matches the user's preferences and communication style depending on social context, improving on the limited, impersonal content and static, predefined style of language rehabilitation systems in previous AAC tools. Combining these two aspects, SocializeChat shows great potential in overcoming the challenges associated with language rehabilitation in aphasia patients.^18^ A range of aphasia patients, including those who have had brain tumours removed, are unable to speak, or have motor impairments, can rely on this new AAC tool to help them with responsive, personalised language rehabilitation training.

SocializeChat can generate multiple sentences of dialogue in real time, provide suggestions tailored to aphasia patients' preferences of topics, and phrase sentences in an appropriate style for the relationship between patients and their conversation partners. SocializeChat's personalised pattern of language communication relies on GPT-4, a core dataset involved in content preference^19^^,^^20^ and social closeness factors.^21^ The aphasia patients' preferences in SocializeChat impact the dialogue suggestions generated by GPT-4, while the degree of social closeness influences the language output.

As the user continuously makes choices from the generated options, the GPT-4 dataset is continuously updated and improved in three ways. Firstly, the dataset utilises preset information entered when using SocializeChat for the first time such as details about the patient, information about family and other relationships, diaries, and personal blogs. Secondly, another input is online information about the patient's interests obtained through GPT-4's internet browsing capability. The third input is the information fed back to GPT-4 during the interaction, allowing the AAC to update and improve continuously. Through these continuous updates, aphasia patients are able to establish a communication pattern with SocializeChat that matches their personal preferences, abilities, and needs.

When patients with oncological aphasia use SocializeChat for language rehabilitation training, they usually need to go through two processes: the initialisation stage and the communication stage. The SocializeChat initialisation phase is mainly based on predefined content preferences and the choice of a social closeness factor, which allows the establishment of a dataset for the patient with oncological aphasia. GPT-4 will provide the preference ratings of patients with oncological aphasia in response to different topics and the reasons behind these evaluations. GPT-4 will also use social closeness to determine a suitable tone for patients with oncological aphasia based on the relationship between the patient and their conversational partner. In this way, these two aspects of information eventually form a comprehensive dataset about the patient. In the second stage, the communication phase, SocializeChat mainly analyses the ongoing conversation and aims to generate appropriate responses. GPT-4 extracts content from the patient's corresponding dataset and determines preference levels, and then generates six sentence suggestions based on the appropriate conversational style for the relevant level of social closeness. Finally, the system presents these generated suggestions as a menu of expressions for the patient.

As an emerging AAC technology, SocializeChat has greatly benefited from the development of LLM text content diversity and the improvement of eye gaze technology. SocializeChat can use GPT-4 to achieve multi-turn conversations to increase the intensity of language training and generate a tailored language rehabilitation training system according to the content preferences of patients with oncological aphasia and the level of social closeness with their partners. In addition, SocializeChat compatibility with eye gaze technology for text input supports accessibility for a wide range of other users with oncological aphasia. At present, the SocializeChat system based on LLM technology can be a good choice for patients with oncological aphasia seeking language rehabilitation therapy. While these potential benefits of SocializeChat are encouraging, many details still require working out.

During the communication phase, SocializeChat is limited to generating six options for dialogue choices, which also limits the precision of the feedback returned to GPT-4. Future work could further optimise the configuration of dialogue options and implement methods of providing more helpful feedback to provide patients with a better language training experience. Another important issue is that even though SocializeChat can build a dataset for each patient using the three types of input described, the system may still be unable to produce what the patient specifically wants in terms of dialogue options. Patients may need to provide a more substantial level of input for SocializeChat to generate more appropriate and preferable sentence suggestions. Future research could explore designing flexible and diverse interfaces that allow patients to correct or override the system if it fails to produce appropriate output in a particular situation.^22^

In conclusion, AAC technology is currently used in the language rehabilitation of patients with oncological aphasia. As existing AAC technology supports speech production, plus utilising a dataset generated by GPT-4, the integration of these two technologies will facilitate real-time social interactions to improve the rehabilitation effects on patients with oncological aphasia.

## CRediT authorship contribution statement

All authors (YZ, QT, SC, and XC) had substantial contribution to either the conception of the work, or the draft of this manuscript. YZ was involved in revising the work. All authors were granted complete access to all the data in the study, with the corresponding author bearing the final responsibility for the decision to submit for publication. The corresponding author affirms that all listed authors fulfill the authorship criteria and that no others meeting the criteria have been omitted. All authors gave final approval of this version to be published.

## Declaration of competing interest

All authors have no conflicts of interest to declare. The corresponding author, Dr. Yingchun Zeng, is a member of the editorial board of the *Asia–Pacific Journal of Oncology Nursing*. The article underwent the journal's standard procedures and was handled independently, with no involvement from Dr. Zeng and their research groups.

## Funding

This study was funded by the 10.13039/501100001809National Natural Science Foundation of China (Grant No. 72004039) to Dr Zeng. The funders had no role in considering the study design or in the collection, analysis, interpretation of data, writing of the report, or decision to submit the article for publication.

## Ethics statement

Not required.

## Declaration of Generative AI and AI-assisted technologies in the writing process

No AI tools/services were used during the preparation of this work.
